# Mechanisms of SARS-CoV-2 and Male Infertility: Could Connexin and Pannexin Play a Role?

**DOI:** 10.3389/fphys.2022.866675

**Published:** 2022-05-23

**Authors:** Temidayo S. Omolaoye, Nour Jalaleddine, Walter D. Cardona Maya, Stefan S. du Plessis

**Affiliations:** ^1^ Department of Basic Sciences, College of Medicine, Mohammed Bin Rashid University of Medicine and Health Sciences, Dubai, United Arab Emirates; ^2^ Reproduction Group, Department of Microbiology and Parasitology, Faculty of Medicine, Universidad de Antioquia, Medellin, Colombia; ^3^ Division of Medical Physiology, Faculty of Medicine and Health Sciences, Stellenbosch University, Tygerberg, South Africa

**Keywords:** SARS-CoV-2, male infertility, connexin, pannexin, cellular communication, gap junction, Blood-testis-barrier

## Abstract

The impact of severe acute respiratory syndrome coronavirus 2 (SARS-CoV-2) on male infertility has lately received significant attention. SARS-CoV-2, the virus that causes coronavirus disease (COVID-19) in humans, has been shown to impose adverse effects on both the structural components and function of the testis, which potentially impact spermatogenesis. These adverse effects are partially explained by fever, systemic inflammation, oxidative stress, and an increased immune response leading to impaired blood-testis barrier. It has been well established that efficient cellular communication *via* gap junctions or functional channels is required for tissue homeostasis. Connexins and pannexins are two protein families that mediate autocrine and paracrine signaling between the cells and the extracellular environment. These channel-forming proteins have been shown to play a role in coordinating cellular communication in the testis and epididymis. Despite their role in maintaining a proper male reproductive milieu, their function is disrupted under pathological conditions. The involvement of these channels has been well documented in several physiological and pathological conditions and their designated function in infectious diseases. However, their role in COVID-19 and their meaningful contribution to male infertility remains to be elucidated. Therefore, this review highlights the multivariate pathophysiological mechanisms of SARS-CoV-2 involvement in male reproduction. It also aims to shed light on the role of connexin and pannexin channels in disease progression, emphasizing their unexplored role and regulation of SARS-CoV-2 pathophysiology. Finally, we hypothesize the possible involvement of connexins and pannexins in SARS-CoV-2 inducing male infertility to assist future research ideas targeting therapeutic approaches.

## Introduction

The discovery, virulence, and transmissibility of severe acute respiratory syndrome coronavirus 2 (SARS-CoV-2) took the world by surprise and have unfortunately led to the ongoing pandemonium. Although some coronaviruses have previously caused epidemics (Hamre and Procknow, 1966; McIntosh et al., 1967; Peiris et al., 2003; Woo et al., 2005), the emergence of SARS-CoV-2 as the virus causing coronavirus disease (COVID-19) has resulted in a pandemic (Huang et al., 2020; World Health Organization, 2020; World Health Organization, 2022).

As of January 28, 2022, SARS-CoV-2 has infected more than 350 million people, with over 5.6 million deaths globally (World Health Organization, 2022). Putting it into perspective, this means that in the space of twenty-five months, the world has lost 0.07% of its population to COVID-19 and 1.6% of the people infected with SARS-CoV-2 globally. This is in part due to the emergence of new variants over time. Up to date, the World Health Organization (WHO) have identified different SARS-CoV-2 variants, which include the alpha variant (B.1.1.7), the beta variant (B.1.351), gamma variant (P.1), delta variant (B.1.617.2), and the currently identified variant, Omicron (B.1.1.529). The most common symptoms of all SARS-CoV-2 variants include fever, sore throat, dry cough, and fatigue. In severe cases, symptoms could include pneumonia or acute respiratory distress syndrome (Taleghani and Taghipour, 2021). SARS-CoV-2 is present in saliva, respiratory fluids, blood, urine, and feces droplets. According to the available clinical data, SARS-CoV-2 not only causes respiratory diseases and affects the lungs but can also induce histopathological changes in various non-respiratory organs and systems, such as the kidney, liver, brain, and heart (Song et al., 2020a; Tan et al., 2020). Seeing that this virus is found in body fluids and could cause abnormality in various systems and organs of the body, the question arose if SARS-CoV-2 is present in the semen and could be transmitted through sexual activity and potentially cause reproductive abnormality.

Stemming from this curiosity, studies began to investigate the effect of SARS-CoV-2 on male fertility. An initial study from Li et al. reported that SARS-CoV-2 was present in the semen samples of acutely infected or recovered patients (Li et al., 2020a). Yang et al. showed that SARS-CoV-2 was present in the postmortem testes of patients with COVID-19 (Yang et al., 2020). Apart from the aforementioned studies, subsequent findings reported SARS-CoV-2 absence from the semen of infected men (Guo et al., 2020; Kayaaslan et al., 2020; Pan et al., 2020). However, all the studies reported that men infected with SARS-CoV-2 displayed abnormal semen parameters, altered hormone profile, and disrupted spermatogenesis. Of interest, a recent study showed the presence of SARS-CoV-2 in the semen sample collected at day 81 since infection from a man who recovered from COVID-19 (Purpura et al., 2021). Although the semen samples of other participants in this study were void of SARS-CoV-2, they all had semen parameter abnormalities, especially asthenozoospermia and oligozoospermia (Purpura et al., 2021). This further highlight that infection with SARS-CoV-2 could adversely affect male fertility. These adverse effects are highlighted and explained in detail in an earlier review article (Omolaoye et al., 2021).

With the understanding that SARS-CoV-2 affects spermatogenesis and sperm quality negatively, the emanating concern is how does this virus exert these detrimental effects observed on male fecundity. These adverse effects are partly explained by the occurrence of fever, hormonal disruption, orchitis, systemic inflammation, oxidative stress, and an increased immune response leading to an impaired blood-testis barrier (BTB). Of note, maintaining efficient cellular communication is required for tissue homeostasis. Cellular communication *via* connexin (Cx) and/or pannexin (Panx) protein channels have been shown to play crucial roles in coordinating cellular communication in different tissues, including the testis and epididymis (Cyr, 2011). Moreover, it has been speculated that these protein channels might have significant involvement in COVID-19 pathogenesis, particularly the Panx; however, the exact role remains unclear. Therefore, the current review will highlight the suggested mechanisms through which SARS-CoV-2 affects male fertility while hypothesizing on the possible involvement of Cxs and Panxs in COVID-19 induced male infertility.

### SARS-CoV-2 and Male Infertility: Mechanisms

SARS-CoV-2 impacts the male reproductive system potentially *via* angiotensin-converting enzyme two receptor (ACE2) utilization as a route to affect reproduction (Youni et al., 2020), similar to its entry to other host cells. Briefly, SARS-CoV-2has four main structural proteins: spike surface glycoproteins (S), small envelope proteins, matric proteins, and nucleocapsid proteins (Harmer et al., 2002; Stanley et al., 2020). The S proteins have two subunits (S1 and S2) responsible for receptor recognition and membrane fusion. SARS-CoV-2 binds its C-terminal domain of the S1 subunit to the host cell’s ACE2 receptor (Illiano et al., 2020). Host proteases such as transmembrane serine protease 2 (TMPRSS2) then cleaves the viral S protein causing a conformational change, allowing permanent fusion of the viral and host cell membranes, and thus infection. SARS-CoV-2 infection elicits a pro-inflammatory cytokine storm, resulting in multi-organ damage such as the lungs, heart, and brain, which are also identified as the main target of SARS-CoV-2 mediated pathogenesis (Selvaraj et al., 2021). The testes of men with COVID-19 are often not exempted from the cytokine storm. The infection causes disrupted spermatogenesis, abnormal semen parameters, and orchitis in these men (Corona et al., 2020; Pan et al., 2020). SARS-CoV-2 may impair male fertility via altered ACE2 expression in the testes, elevated body temperature (fever), pro-inflammatory cytokines storm, hormonal disruption, oxidative stress, or possibly through the functional disruption of Cxs and/or Panxs.

### ACE2, SARS-CoV-2, and Male Infertility

ACE2 is a transmembrane zinc metallopeptidase containing a single catalytic domain with homolog to angiotensin-converting enzyme (ACE), attached to the cell membrane of cells in different tissues, including the testis (Li et al., 2020b; Wang et al., 2020). The abundant expression of ACE2 in the different cells of the testes (spermatogonia, Leydig cells, and Sertoli cells) and some other components of the male genital tract such as the prostate gland and epididymis (Song et al., 2020b) have brought about the concept that SARS-CoV-2 can potentially affect male fertility negatively. Furthermore, ACE2 expression in the Leydig cells has been shown to aid testosterone production and steroidogenesis regulation, and it mediates local vascular regulation to balance the interstitial fluid volume. Ang1-7 and Mas, which are products of ACE2 catalyzation, were also observed in the seminiferous tubules and the interstitial component of the seminiferous tubules of men with normal spermatogenesis, but not in infertile men (Reis et al., 2010). Additionally, the protease that aids the binding and subsequently allows the entry of SARS-CoV-2 is abundantly expressed in the testes (Liu et al., 2020; Seymen, 2021). Sine ACE2 and TMPRSS2 are present in the male genital tract, including the testes, it can be speculated that upon viral entry, male fertility could be impaired.

### SARS-CoV-2, Fever, and Male Infertility

Another concern about how SARS-CoV-2 may affect male fertility is sustained fever. Recent studies have indicated that high body temperature may induce a secondary cytokine storm (hyperinflammatory syndrome with fulminant and fatal hypercytokinemia with multiorgan failure) (Batiha et al., 2020; Swayne et al., 2020) which in turn harm spermatogenesis in COVID-19 patients (Batiha et al., 2020; Paoli et al., 2020). Hence, abnormal changes in the cytokine profile may affect male fertility, with a long-term impact.

Although the testis contains heat shock proteins (HSP) that protect against heat and chemical radiation, excessive exposure could harm male reproduction. Studies have revealed that spermatocytes are the most susceptible to heat shock, and apoptosis of these cells occurs upon exposure to heat (Rockett et al., 2001). Rockett et al. reported that specific proteins were upregulated following heat shock while others were downregulated. The subsequent effect of fever on male reproduction includes a temporary reduction in relative testicular weight and a brief period of partial or complete infertility (Rockett et al., 2001). In addition, a decrease in sperm motility, reduced *in vitro* fertilization (IVF) rate by sperm from heat shock (Jannes et al., 1998), increased rate of embryonic degeneration in rats (Setchell et al., 1988) and rams (Mieusset et al., 1992) were also reported.

Briefly, during heat shock, there is induction of HSPto protect against the damaging effects of heat. However, upon prolongation, the rate of cell cycle disruption is higher than the produced HSP, hence inducing apoptosis. Apoptosis ensues because there is a reduction in the expression of anti-apoptotic genes such as Bag-1 to protect against apoptosis. The reduction of Bag-1 may also allow the inappropriate HSP-mediated protein refolding during the cell recovery phase from stress. The abnormally folded proteins could, in turn, induce apoptosis of the affected cells. The following receptors were upregulated in response to heat shock: laminin receptor, laminin γ2, and HSP25 (Rockett et al., 2001). Laminin, along with collagen type IV, is a major component of the peritubular basal lamina of the seminiferous epithelium (Santoro et al., 2000). Interactions between Sertoli cells and the basal lamina are essential for maintaining the BTB.

Additionally, SARS-CoV-2 infection could enhance inflammatory response leading to fever. Human monocytes infected with SARS-CoV-2 exhibit increased production of chemokines (IL-8 and CXCL10), inflammatory cytokines (IL-6, TNFα and IL-10) and molecules that are generated primarily through an oxidative pathway from arachidonic acid (AA), including leukotrienes (the cysteinyl leukotriene -CysLT- and leukotriene B4 -LTB_4_-) (da Silva Gomes Dias et al., 2020). Other metabolites of AA, such as prostaglandin E2 (PGE2) are elevated in patients with COVID-19 disease and are associated with the disease severity and sex, with higher levels in men compared to women (Ricke-Hoch et al., 2021).

Moreover, in male zebrafish, PGE2 was demonstrated to reduce the proliferation of spermatogonia progenitors, although it increased the self-renewal proliferation of type A undifferentiated spermatogonia resulting in their accumulation (Crespo et al., 2020), with Cx43 serving as important portals for prostaglandin release (Riquelme et al., 2015). Therefore, the controlled prostaglandin levels released during COVID-19 could be a critical factor that induces the sperm alteration observed in some men infected with SARS-CoV-2.

### SARS-CoV-2, Inflammation and Male Infertility

During a viral infection, there is an elevation in systemic inflammation. This inflammatory response induction is meant to protect the host; nevertheless, the prolonged or sustained response could potentially cause harm (Hassanin et al., 2018). The mediation of systemic inflammation on somatic cells is different from the immune response in the testes. This is due to the BTB near the basement membrane of the seminiferous tubules, where it divides the epithelium into the basal and adluminal compartments. The BTB elevates the immune status in the testes and preserves male gonads from uncontrolled immune responses. However, the overproduction of inflammatory cytokines induced by viral infections can lead to autoimmune responses and infiltration of leucocytes, which subsequently disrupt spermatogenesis and interfere with sex-related hormones secretion (Hedger and Meinhardt, 2003). During a sustained inflammation state, there is an increase in the generation of inflammatory cytokines and transcriptional factors such as interleukins (IL)-1, IL-6, IL-8, IL-10, IL-17, TNF-α, and NF-kβ, which in turn leads to an increase in the production of reactive oxygen species (ROS) and concomitantly elevating polymorphonuclear leukocytes and granulocytes. Although, a specific timeline for the initiation of each inflammatory response is yet to be agreed upon; however, studies have shown that the intensity, and the time course of the observed inflammatory response (s) and or subsequent impact depends on the cell or tissue types, the concentration of the pro-inflammatory agent that the cell or tissue of interest were treated with, the duration of treatment. These responses consequently lead to the development of oxidative stress (OS). The occurrence of OS may cause impaired spermatogenesis by damaging the intactness of BTB or disrupting testicular cells functions (Haghpanah et al., 2021). In severe cases of COVID-19, it was shown that there is an increase in the level of expression of inflammatory cytokines and their receptors in testicular cells (Shen et al., 2020). This suggests that systemic inflammation may in part disrupt the integrity of the BTB. Thus, causing its permeability to inflammatory cytokines and consequently lead to the altered spermatogenesis seen in these cases.

### SARS-CoV-2, Altered Hormone, and Male Infertility

SARS-CoV-2 can cross the blood-brain barrier (BBB) and dock with ACE2 expressing neurons and glial cells, thereby causing neuroinflammation and neuropathogenesis. The hypothalamus, which is the center that controls body temperature and hormonal balance, amongst other physiological functions, is therefore affected during SARS-CoV-2 infection (Wu et al., 2020). Studies have also shown that SARS-CoV-2 is able to cross the BBB through a transcellular pathway accompanied with matrix metalloproteinase 9 (MMP9) mediated basement membrane disruption without causing tight junction alteration (Zhang et al., 2021).

Under normal circumstances, the hypothalamus secretes the gonadotropic releasing hormone (GnRH), and it activates the release of follicle‐stimulating hormone (FSH) and luteinizing hormone (LH) from the anterior pituitary. The secreted FSH stimulates the Sertoli cells to nurture maturing germ cells, while LH stimulates Leydig cells towards testosterone production. This feedback loop is strictly controlled. However, any condition that can cause the malfunctioning of the hypothalamic‐pituitary‐gonadal axis (HPGA) may result in male fertility impairment. For instance, low GnRH levels cause a decrease in FSH and LH, resulting in impaired function of the Sertoli and Leydig cells. Although there was no significant difference in the serum testosterone and serum FSH, the ratio of testosterone to LH (T:LH) and the ratio of FSH to LH (FSH:LH) were significantly reduced in the sera of COVID-19 patients compared to the control group. This suggests that being infected with SARS-COV-2 may alter sex-related hormones (Ma et al., 2020). Additionally, infection with COVID-19 has been shown to cause histopathological changes in postmortem testicular tissues (Yang et al., 2020). Taken together, it is theoretically possible that the normal functioning of the HPGA is altered during SARS-CoV-2 infection, which subsequently could cause Leydig and Sertoli cell malfunctioning, thereby driving the pathogenesis of testicular dysfunction in COVID-19 patients.

Having discussed the already suggested pathways through which SARS-CoV-2 can negatively impact male fertility, the remaining section will highlight the potential role of Cx and Panx in SARS-CoV-2 induced male infertility.

### Connexins and Pannexins: The Communicating Channels

Tissue homeostasis is dependent on signal transduction that regulates intimate molecular exchanges between extracellular, intracellular, and intercellular networks. Direct cell-to-cell contact or interaction between cells and their extracellular environment is crucial in maintaining proper cellular functions. Such communication is maintained *via* Cxs or gap junction (GJ) channels (Raghavan et al., 2021; Taylor et al., 2021) or *via* pore-forming channels -Panxs. The functional properties of these channels are attributed mainly to different physiological and pathophysiological stimuli and their channel activity and localization, which are evidenced to be modulated by different post-translational modifications (Luu et al., 2021).

Cxs are specialized vertebral intercellular channels widely expressed by many mammalian cells (Swayne et al., 2020). To date, 21 Cx isoforms have been identified in humans, while 20 isoforms have been described in mice; 25 to 62 kDa in size and abbreviated according to their molecular weight (as an example: Cx43, is a Cx with 43 kDa in size) (Jalaleddine et al., 2019; Kazan et al., 2019; Malik and Eugenin, 2019). Some Cxs are tissue-specific, while others are expressed abundantly and ubiquitously relative to their function (Beyer and Berthoud, 2018; Valdebenito et al., 2018; Ahmadian et al., 2019). On the other hand, Panxs -the novel transmembrane protein channels-comprises of only three members: pannexin-1 (Panx1), pannexin-2 (Panx2), and pannexin-3 (Panx3); denoted as PANX1, PANX2, and PANX3 in humans (Crespo Yanguas et al., 2017; Okamoto and Suzuki, 2017). Panx1 has been reported to be ubiquitously expressed in most mammalian tissues, including the testis, ovary, placenta, and prostate (Willebrords et al., 2016; Begandt et al., 2017; Okamoto and Suzuki, 2017), while Panx2 and Panx3 expressions are more restricted to brain, skin, and bone (Diezmos et al., 2016; Freund-Michel et al., 2016; Jiang and Penuela, 2016; Willebrords et al., 2016; Begandt et al., 2017; Okamoto and Suzuki, 2017).

**FIGURE 1:**
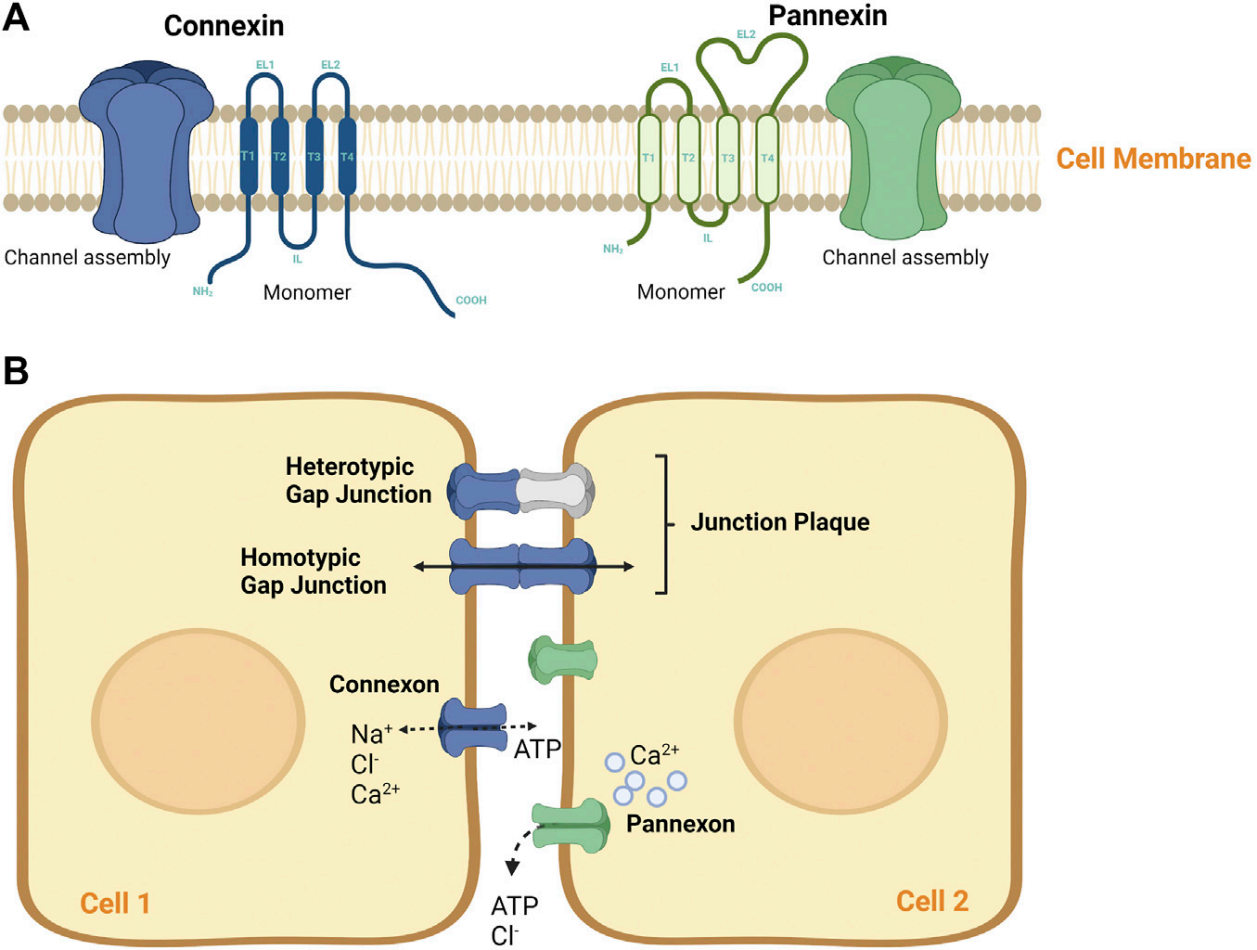
Connexin and pannexin molecular structure. **(A)** A schematic diagram of Connexin and Pannexin protein structure. Both protein channels share similar topology by having four α-helical transmembrane (T) domains, two extracellular loops (EL), and one intracellular loop (IL), with their amino (NT) and carboxyl termini exposed to the cytoplasm. Both, Cxs and Panxs are shown to oligomerize into a hexamer to form single membrane channels at the cell surface, namely **(B)** A representation of Connexons and Pannexons in cellular communication. Connexons may mediate autocrine signaling as independent hemichannels or may form junctional plaque (or gap junction) via the docking of two connexons of adjacent cells. Connexons can be of homomeric hemichannels (single Cx isoform) or heterotypic hemichannels (multiple Cx isoforms), that can interact with each other in different ways to mediate intercellular communication. Pannexons are known to mediate paracrine signaling. These channels tend to mediate paracrine signaling, however, they do not form GJ plaque.

While no sequence homology exists between Cxs and Panxs, they share similar topologies. Both protein families display four α-helical transmembranes (TM) domains, two extracellular loops (EL), and one intracellular loop (IL), with their amino (NT) and carboxyl termini exposed to the cytoplasm (Wei et al., 2015; Waisman et al., 2015). Unlike Cxs, which mediate autocrine signaling by forming a 2–3 nm gap separation via the docking of two “connexon” hemichannels (Waisman et al., 2015), Panxs channels do not form gap junctions (GJs) (Figure 1). Panxs are integral large pore-forming channels that mediate paracrine signaling *via* ATP release into the extracellular milieu (Penuela et al., 2014; Draganov et al., 2015). Analogous to Cxs, Panxs also function as conduits of physiologically essential molecules and ions such as intracellular Ca2+ as being a secondary messenger, glucose uptake, and other small molecules (up to 1.5 kDa). Nonetheless, the mediated autocrine and paracrine cell signaling plays diverse roles in achieving proper regulation and coordination of different tissue functions. Regulatory mechanisms include vascular functions and permeability, smooth muscle proliferation, platelet aggregation, and leukocyte transmigration (Langlois et al., 2014; Ma et al., 2014; Makarenkova and Shestopalov, 2014). Of note, the role of Panx channels has been well implicated in the development of several tissues and cell types, including cell proliferation and differentiation (Oviedo-Orta et al., 2013; Pfenniger et al., 2013; Wang et al., 2013; Bond and Naus, 2014). Overall, these channels need to be tightly regulated to maintain cellular homeostasis.

### Connexins and Pannexins in Inflammation: Relative to COVID-19

Inflammation is a disease hallmark, requiring immune system activation, accompanied by regeneration, apoptosis, or necrosis events. The immune response to an injury requires ATP release into the extracellular space for inflammasome maturation (Orellana et al., 2013), which is reflected by the production of inflammatory cytokines and chemokines (Orellana et al., 2013). Together, Cxs and Panxs play a critical role during the inflammatory response, highlighted in this section.

Among the Panx protein family, Panx1 has been the best characterized protein channel in diseases for ATP release (Gao et al., 2013; Hung et al., 2013). Panx1 is believed to be a key regulator of the inflammatory process by forming a complex with the purinergic receptors (P2X1, P2X4, and P2X7), thus mediating ATP release that leads to pro-inflammatory cytokine processing through the maturation and release of IL-1α, IL-1β, and IL-18 (Penuela et al., 2012; Looft-Wilson et al., 2012; Lohman et al., 2012; D'hondt et al., 2013). Such protein complex formation has also been shown to generate ROS (Kerr et al., 2012). In addition to inflammasome activation, Panx1 mediated ATP release channels have also been attributed to immune functions through the “find-me” signals that play a role in removing apoptotic cells (Seminario-Vidal et al., 2011; Cerami et al., 2012; Gulbransen et al., 2012; Kerr et al., 2012; Looft-Wilson et al., 2012). Furthermore, the Panx1-purinergic complex contributed to cell death under ischemic conditions and seizures. This was studied in different diseases, such as inflammatory bowel diseases, Crohn’s, AIDS, and epilepsy (Santiago et al., 2011; Pointis et al., 2011; Ishikawa et al., 2011; D'hondt et al., 2011; Bond et al., 2011; Billaud et al., 2011; Bargiotas et al., 2011). Of note, Panx1 channel expression and activity were reported to increase significantly upon injury or inflammation (Swayne et al., 2010). Besides inflammation induction, the ATP released *via* Panx channels into the extracellular environment has also been associated with tumor growth and cancer metastasis, thus promoting cancer progression (Garré et al., 2010; Iwamoto et al., 2010; Riteau et al., 2010). This was addressed by Furlow et al., who showed that breast cancer cells expressing the mutant form of Panx1 (Panx11–89) increase ATP release, thus enhancing the metastatic potential of these cells (Scemes et al., 2009; Celetti et al., 2010; Chekeni et al., 2010; Furlow et al., 2015). Similarly, Panx2 and Panx3 have also been shown to be a conduit for ATP release; however, their functions were mainly attributed to cellular differentiation events (Penuela et al., 2009; Figueroa and Duling, 2009). Taken together, findings support the involvement of Panxs in inflammasome activation under “non-homeostatic” conditions.

Several other studies also highlighted GJ-mediated cell communication's importance in regulating inflammation in different diseases, including atherosclerosis, hypertension, and diabetes (Brough et al., 2009; Dbouk et al., 2009). However, evidence also suggested that Cxs are involved in inflammation as independent functional hemichannels and not as GJs (Willebrords et al., 2016). It was evidenced that ischemic injuries induced inflammation results in the opening of Cx hemichannels (Thompson et al., 2008). Changes in Cx expression and GJ coupling and functioning in response to systemic inflammation have also been evidenced. For example, in response to inflammation, Cx40 and Cx37 expressions were downregulated during pulmonary diseases (Pelegrin, 2008). In another study, the expression of Cx43 and Cx32 and their GJ coupling were inhibited in astrocytes and liver diseases. Such response was due to releasing the pro-inflammatory cytokine IL-1β (Kang et al., 2008; Pelegrin, 2008). Analogous to Panxs, Cxs are involved in ATP release. For example, Cx43, being the ubiquitously expressed hemichannel among the Cx protein family, has been shown to participate in ATP release in many cell types (Penuela et al., 2007; Yen and Saier, 2007). It is suggested that ATP release is modulated by the activity of Cx43 hemichannels (Locovei et al., 2006; Eugenín et al., 2007). On the contrary, Cx43 as a functional GJ has protective roles, as seen in different types of cancer (Fischer et al., 2005; Eltzschig et al., 2006). Using *in vivo* and *in vitro* approaches, the enhancement of Cx43 expression and GJ coupling resulted in the regulation of several cytokines (Moreno, 2004; Söhl and Willecke, 2004). Therefore, understanding these channels’ expression profiles and function in inflammatory conditions makes them an attractive therapeutic potential to help manage different diseases.

### Connexins and Pannexins in COVID-19 Pathology

Despite the extensive research on understanding SARS-CoV-2 viral mechanism and vaccine evaluation, this pandemic is still spreading worldwide (Bennett and Zukin, 2004). Till date, there is still no clear evidence about the interaction between SARS-CoV-2 and the Cxs and Panxs protein channels. Since Panx1 channels are critical regulators of the inflammasome, playing a crucial role in lung vascular inflammation and edema (Baranova et al., 2004) and host responses to viruses (Sáez et al., 2003; Bao et al., 2004). It is crucial to consider Panxs’ involvement in the SARS-CoV-2 inflammatory mechanism. A recent study by Luu et al. has shed light on SARS-CoV-2 inducing Panx1 channel activation. Interestingly, this was associated with ATP, and IL-1β increased mRNA expression (Bennett and Zukin, 2004). Although more investigations are required, these findings provide a rationale in supporting further investigations regarding the involvement of Panx channels in SARS-CoV-2. Nonetheless, Cxs involvement should also be addressed. It is observed that the expression of Cxs, specifically Cx43, in addition to adherents junction proteins, are significantly decreased upon infection with SARS-CoV-2. This was explained by Raghavan et al., who showed that the S protein of the virus induced degradation of GJ coupling, thus affecting endothelial barrier function, which resulted in vascular damage and loss of barrier integrity, as well as disrupted intercellular communication, as observed in COVID-19 individuals (Goodenough and Paul, 2003). Moreover, it is important to note that pro-inflammatory cytokines decrease Cx43 expression (González et al., 2002). Both Cxs and Panxs provide a promising approach for treating inflammation, as they are attractive targets to consider in understanding COVID-19 pathogenesis. Although several pertinent queries remain to be elucidated regarding their induced disease mechanisms, particularly in SARS-CoV-2 induced male infertility, this study will hypothesize their plausible involvement.

### SARS-CoV-2, Communicating Channels, and Male Infertility

As mentioned earlier, GJs are intercellular plasma membrane channels that create electric and metabolic coupling, mediating cell-to-cell communication in a wide variety of tissues. They are involved in embryogenesis, spermatogenesis, and other cellular processes (Cyr, 2011).

In spermatogenesis, the development and differentiation of cells require strict regulated cell-to-cell interaction, rendering the GJs channels important to the process of spermatogenesis. The importance of junctional channels for male fertility cannot be overemphasized. For instance, loss of Cx43 in mouse fetus leads to a decrease in the number of germ cells (Kidder and Cyr, 2016), while loss of Cx43 in adults leads to the absolute inhibition of spermatogenesis. Cxs 26, 32, 33, 36, 45, 46, and 50 are present in different testis cell types in various mammals, making them essential for differentiation and maturation. Loss of Cx46 leads to increased germ cell apoptosis and loss of BTB integrity (Gregory et al., 2011). Loss of Cx43 in the Sertoli cells leads to Leydig cell hyperplasia, suggesting a connection between the 2 cells (Sharpe et al., 2003; Lie et al., 2013). In the epididymis, Cxs, 26, 30, 3, 31.1, 32, and 43 are identified and involved in the epithelium differentiation. The decrease in Cx43 leads to decreased sperm motility (Dubé and Cyr, 2013). Different Cxs can form heteromeric connexons, and connexons of different connexin compositions can dock to form individual GJ. Building on these facts, the number of different types of connexin channels in the testis is likely to be larger than the number of Cxs present (Laird, 2006).

A study reported the decreased Cx43 expression in azoospermia (Adam and Cyr, 2016) and a decline in the mRNA Cx43 expression of men with non-obstructive azoospermia (Dubé et al., 2012). Another study reported a correlation between the severity of seminiferous tubule damage and the decrease in Cx43 immuno-staining (Kotula-Balak et al., 2007). Although these effects are seen in azoospermic men, no exact mechanism can be pinpointed as the original pathway affected, and it cannot be said that the symptoms of diseases resulted from the loss, decreased or mal expression of Cxs. However, what is valid is that these communicating channels are essential for spermatogenesis, either during fetal or adult life.

In addition to the importance of endocrine control and other maintained physiological processes, the testicular cell-cell interactions in spermatogenesis are also vital (Pointis et al., 2010). GJs are localized between adjacent Leydig cells, between Sertoli cells, and between Sertoli cells and specific germ cells. The cell-cell interaction is accomplished through the dependent junctional pathways necessary for regulating spermatogenesis and maintaining male reproduction (Mruk and Cheng, 2004). The junctions are composed of proteins implicated in cell adhesion (catenin and nectin), regulation of paracellular diffusion, establishing cell polarity, and cell attachment such as the BTB (occludin and claudin) and GJ (Cxs) (Pointis et al., 2010).

A study that investigated the effect of SARS-CoV-2 on BTB of patients who died from COVID-19 complications revealed an increase in testicular inflammation and the destruction of the BTB (Peirouvi et al., 2021). Significant adverse changes in the arrangement of testicular cells and decreased Sertoli cells number were observed. Furthermore, a significant reduction in testicular occludin, claudin-11, and Cx43 expression were also detected in the COVID-19 patients (Peirouvi et al., 2021).

On the other hand, Panx-1 and Panx-3 are expressed in the testis and epididymis (Cyr, 2011). Panx-1 is mainly expressed in the Sertoli cells at the level of the BTB, while Panx-3 is localized in the interstitial space, specifically found on Leydig cells. Panxs are also present in the epididymis and may mediate the release of ATP into the epididymal lumen to aid sperm maturation. Thus, the basal presence of Panx-1 in the epididymis may regulate principal cell function and mediate the secretion of ATP into the basal extracellular space (Cyr, 2011).

**FIGURE 2:**
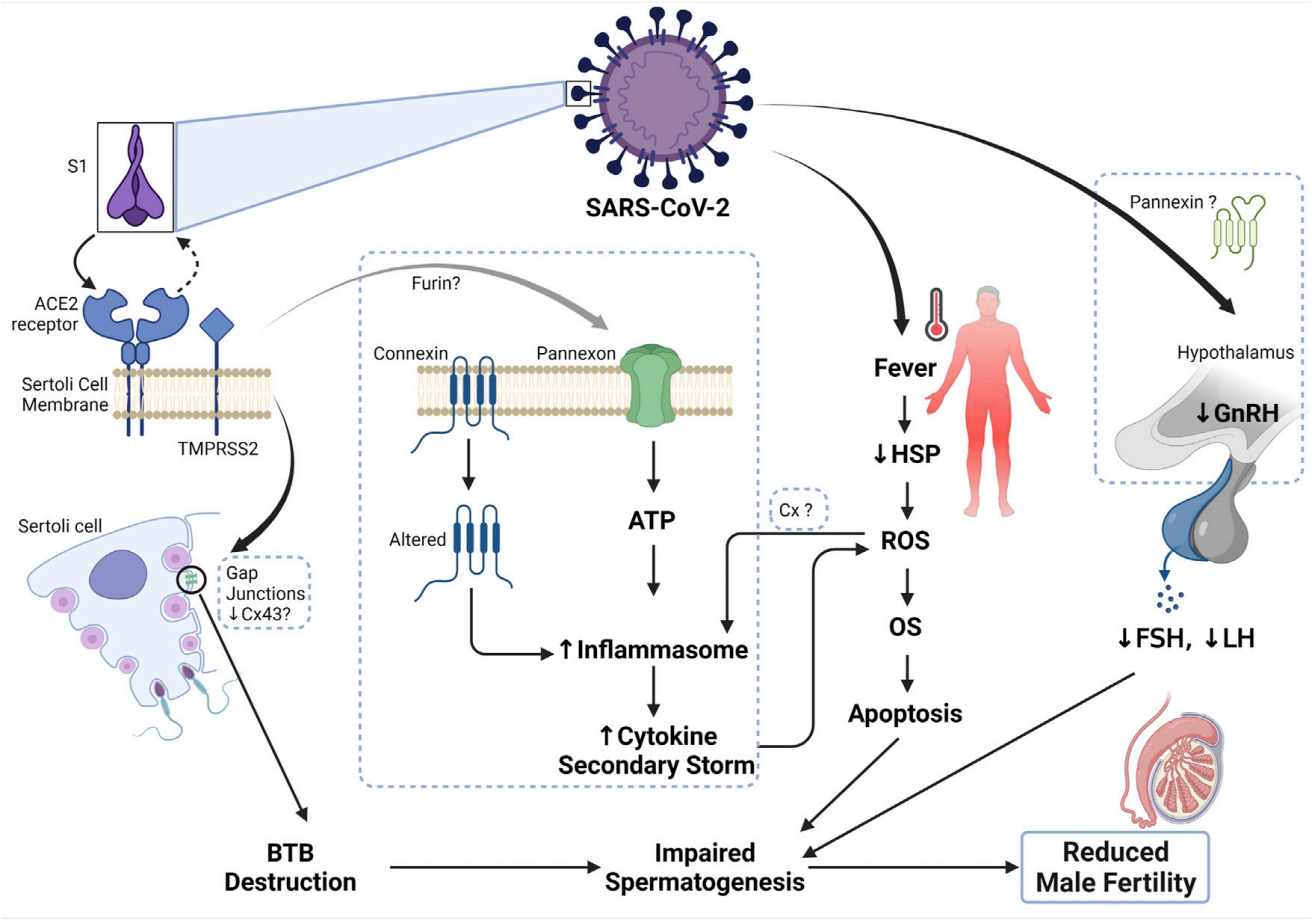
Mechanism of SARS-CoV-2 and male infertility. The S1 subunit of the SARS-CoV-2 binds the ACE2 receptor on the Sertoli cell membrane, which in turn activates TMPRSS2 and cause a conformational change to the complex, thus allowing the entry of the virus. Connexin 43 is abundant on the Sertoli cell membrane and has been shown to play a role in the Sertoli cell’s functionality. In the state of inhibition or depletion of Cx43 on Sertoli cells due to infection or apoptosis, spermatogenesis was reported to be completely inhibited. Therefore, upon SARS-CoV-2 entry, there is a decrease in the expression of Cx43 and other junctional proteins, thus disrupting the blood-testis barrier, and subsequently impairing spermatogenesis. Additionally, the binding of SARS-CoV-2 to ACE2 receptor could plausibly cause the opening of the pannexin channel, a process dependent on furin (another protease that aids viral entry), which could cause the release of ATP into the cell. Excessive ATP leads to the formation of inflammasome and thus cytokine secondary storm ensues. Elevated inflammation inturn causes an increase in the formation of reactive oxygen species (ROS) and further leads to the development of oxidative stress (OS). Occurrence and sustained OS status causes apoptosis and subsequently impaired spermatogenesis. Persistent fever can also increase the generation of ROS and cause OS, due to the reduction of heatshock proteins (HSP) during viral infection. Since SARS-CoV-2 can cross the blood-brain barrier, upon infection, the function of the hypothalamus is impaired, thus resulting in reduced secretion of gonadotropin-releasing hormone and consequently reducing the generation of luteinizing (LH) and follicle-stimulating hormones (FSH). Altered endocrine control leads to impaired spermatogenesis and ultimately, reduced male fertility.

Although studies have shown that Panx-1 channels are inactive under healthy conditions, these channels are rendered actively open under diseased conditions, thus propagating the pathogenesis of the disease (Valdebenito et al., 2018; Gajardo-Gómez et al., 2020). This particular observation has not yet been reported in the testis and epididymis. Though what is known is that Panx-1 is strategically localized on the Sertoli and Leydig cells of the testis. This means that 1) they may play a role in the GJ complexes of the BTB, thereby allowing the influx of small ions, nucleotides, and proteins, which may subsequently aid the release of ATP. 2) Their localization in the Leydig cell may indicate their use for testosterone production. This is mainly explained by a study performed on orchidectomized rats. Studies on the epididymis have indicated that orchidectomy increases the number of Panx1 and Panx3 immunoreactive bands, as well as of total protein levels throughout the epididymis, while testosterone maintenance with orchidectomy reduces the number of bands in the caput and corpus; suggesting that testosterone is required for the post-translational function of Panx-1. Taken together, it is unclear whether the opening of the Panx-1 channel is detrimental to spermatogenesis and sperm maturation, as seen in other systems. Nevertheless, what is valid is that the activation of Panx-1 during infection may pose a threat to spermatogenesis and consequently male fertility. In summary, since ACE2, Panxs, and the purinergic receptors are present in the testis and epididymis (Lee et al., 2000; Illes et al., 2020), upon SARS-CoV-2 infection; theoretically, Panx-1 present in the Sertoli and Leydig cells would be opened and cause the activation of the purinergic receptors; which could propagate the inflammation signaling. When inflammation is prolonged, male fertility is thus impaired. The mechanisms through which SARS-CoV-2 may affect male fertility are summarized in Figure 2.

### Connexins and Pannexins as Therapeutic Targets in Male Infertility and SARS-CoV-2: A Commentary

Considerable insights continue to emerge regarding Cxs and Panxs molecular mechanisms in different diseases; however, developing new intervention strategies using drug therapy that targets Cx and Panx channels may prove beneficial. This will also bridge the gap between Cx and Panx therapeutic strategies with COVID-19 management and treatment response.

Drug targets are available to inhibit channels of Cx and Panx proteins. These include alcoholic substances, glycyrrhetinic acid, anaesthetics, and fatty acids. It is important to note that such pharmacological tools may not differentiate between GJ or hemichannels and, therefore, might require the need of a specific targeting inhibitor. In this case, using mimetic peptides, which reproduce specific amino acid sequences in either Cx or Panx, might serve as a beneficial approach (L. Harris, 2001; Ware and Matthay, 2000), yet novel therapies are urgently required. In the context of male infertility, therapeutic strategies have to focus on either the re-expression of the Cxs or on enhancing Cx hemichannel activity to retain its proper junctional coupling, which restores disrupted intercellular communication (Panchina et al., 2000). Also, targeting strategies should not exclude the Panx protein channels. Probenecid, a potent Panx-1 inhibitor, has proven to be a promising target of inflammation in different diseases (Beyer et al., 1995; Goodenough et al., 1996; Duffy et al., 2000), including COVID-19 pathogenesis, where Panx-1 mRNA expression was responsible for increased ATP and IL-1β levels in human lung epithelial cells infected with SARS-CoV-2. Another FDA-approved drug, spironolactone, has also proven to be a potential Panx-1 blocker in COVID-19 patients. Knowing that the drug is meant for hypertension patients, spironolactone could be beneficial as a Panx-1 blocker in the context of COVID-19 (Aoumari et al., 1990; Bennett and Zukin, 2004).

## Conclusion

The current evidence does not allow to assure the role of Cxs and Panxs in the adverse reproductive effects observed in individuals with COVID19, however, there are some indications of this relationship, providing a justification that supports the need for new studies evaluating the role of these protein channels, with the purpose to understand and perhaps avoid, through the use of blockers of these proteins as treatments, guaranteeing that human fertility will not be affected due to SARS-COV-2; an infection that is here to stay.

Based on current evidence, plausibly SARS-CoV-2 is not present in semen. Nevertheless, it exerts a negative effect on gametogenesis, a process that subsequently affects sperm parameters, guaranteeing that the testicular architecture could be altered. Therefore, it is essential to understand the role of these protein forming hemichannels in the pathogenesis of SARS-CoV-2 induced male reproductive impairment, which could help avoid these harmful effects.

Finally, infertility cases due to male factors continue to increase worldwide, and after the pandemic, this might further increase. Therefore, understanding these possible interactions will allow bridging critical knowledge gaps in our perception of the molecular mechanisms involved in male infertility.
